# Predictive factors of hesitancy to vaccination against SARS-CoV-2 virus in young adults in Spain: Results from the PSY-COVID study

**DOI:** 10.1016/j.jvacx.2023.100301

**Published:** 2023-04-18

**Authors:** Corel Mateo-Canedo, Juan Pablo Sanabria-Mazo, Laura Comendador, Juan Sebastián Rojas, Meritxell Carmona, Neus Crespo-Puig, Fiorella Anyosa, Clara Selva, Albert Feliu-Soler, Narcís Cardoner, Juan Deus, Juan V. Luciano, Jorge Luis Méndez-Ülrich, Antoni Sanz

**Affiliations:** a Universitat Autònoma de Barcelona, Spain; b Parc Sanitari Sant Joan de Déu, Spain; c Hospital de la Santa Creu i Sant Pau, Spain; d Universitat Oberta de Catalunya, Spain; e Universitat de Barcelona, Spain

**Keywords:** COVID-19, Hesitancy, Reluctance, Vaccination, Attitudes, Beliefs

## Abstract

Widespread population vaccination against the SARS-CoV-2 virus is a matter of great interest to public health as it is the main pharmacological measure to contain the COVID-19 pandemic. Hesitancy/reluctance to vaccination has become a main barrier to containing the pandemic. Young adults are the age group with the greatest resistance to vaccination, even in countries with the highest vaccination rates during this pandemic. The objective of this study was to identify the main predictive factors of vaccination intention and profile people with hesitancy/reluctance to vaccinate against SARS-CoV-2 virus in young adults living in Spain during the pandemic. A cross-sectional study was conducted based on the administration of an online survey (PSY-COVID-2) that evaluated the intention of vaccination together with a wide range of sociodemographic, social, cognitive, behavioral and affective variables in a sample of 2210 young adults. 14% of the sample showed hesitancy/reluctance to vaccination at the beginning of their vaccination campaign. A total of 35 factors were associated (small to medium effect sizes) with the intention to get vaccinated. A reduced set of 4 attitudinal and social variables explained 41% of the variability in vaccination intention: attitude to the vaccination, trust in health staff/scientists, conspiracy beliefs about SARS-CoV-2 and time spent being informed about COVID-19. These variables showed good sensitivity/specificity for classifying people as reluctant/not reluctant to vaccination, properly classifying 86% of people. Psychosocial processes related to attitudes, trust and information are the main predictors of vaccination intention in a highly reluctant group such as the young adult population.

## Highlights

• 14% of Spanish young adults showed hesitancy/reluctance to vaccination against the SARS-CoV-2.
• Main predictors of vaccination intention were attitudes, trust, and information.
• Main predictors accounted for 41% of the variability regarding intention to get vaccinated.
• Main predictors made it possible to detect vaccination intention with 86% accuracy.
• Adequate management of public information is critical to manage the hesitancy to vaccination in young adults.

## 1 Introduction

Vaccination has historically greatly reduced the effect of infectious diseases and is generally safer and more effective than curative drugs [[1], [2]]. The benefits of vaccination transcend the prevention of infection, morbidity and mortality of people, because it also contributes to the reduction of the costs for the public health system and promotes the disappearance of new resistant strains [3]. In turn, vaccination provides social benefits, such as indirect protection to people that cannot be vaccinated (due to age, chronic diseases, etc.), while also combating the socioeconomic inequity of access to health compared to other types of pharmacological treatments [[3], [4]].

The *World Health Organization* (WHO) [5], stated that one of the biggest risks to preventing the spread of disease is hesitation over vaccination. This phenomenon underlies a set of negative beliefs, attitudes, and behaviors regarding vaccination. Previous studies indicate that resistance to vaccination is sustained despite its proven success against common and serious diseases [3].

The development and mass administration of vaccines is seen as crucial to stop the spread of the SARS-CoV-2 virus, as well as to achieve herd immunity and curb the emergence of new variants of the virus. Given the characteristics of the virus and the vaccines administered, it is necessary that between 80 and 90 % of the population be vaccinated to achieve herd immunity [[6], [7]]. The lack of precedent for an international health emergency such as the COVID-19 pandemic contributes to the fact that information strategies on the virus and the implementation of the protection and prevention measures have not followed a pre-established action plan [8]. In turn, given its novelty and accelerated production process, widespread initial uncertainty has emerged regarding the efficacy and side effects of the vaccine. Although currently available vaccines have been shown to be safe and effective, especially for the prevention of serious cases, hospitalization, and death, there is a sizable section of the world's population that is reluctant to be vaccinated [5].

At a time when new variants of SARS-CoV-2 are continuously emerging, the need to achieve a global level of immunity is highlighted, and it is critical for global public health to identify resistant population groups as well as the main barriers associated with hesitance/reluctance to vaccination. Several studies have analyzed the predictors of resistance to vaccination. There is some consensus regarding the sociodemographic characteristics that predict vaccination resistance: being young [[9], [10]], being male [[10], [11], [12]], a low level of education and income [[9], [10]], a conservative political ideology [[9], [10], [13]], being black [[13], [14], [15]] and not having received the flu vaccine [[13], [16]]. Other studies have identified psychosocial factors related to hesitance to vaccination, such as perceived low vulnerability to illness [[12], [13], [17]] or low media confidence [18].

Although some studies [[19], [20], [21]] have indicated that the young population showed high levels of vaccination intention before access to vaccines available to them, the reports in Spain and in the rest of the world [23] highlight that people aged 18 to 49 are the adult population with the lowest vaccination rate. Therefore, it is necessary to analyze in a global perspective the factors related to the intention to get vaccinated in this age range.

This research presents an exploratory approach, seeking to identify the predictive power of affective, cognitive, behavioral, social, and sociodemographic factors of vaccination intention in a sample of young adults in Spain. Unlike previous studies, focused on a specific and reduced set of predictive variables (sociodemographic characteristics, specific personality traits, media, etc.), the assessment tool used in the present study was addressed to collect a broad spectrum of potential predictors which could be related to deciding whether to get vaccinated against SARS-CoV-2.

## 2 Method

### 2.1 Design and procedure

**Fig. 1:**
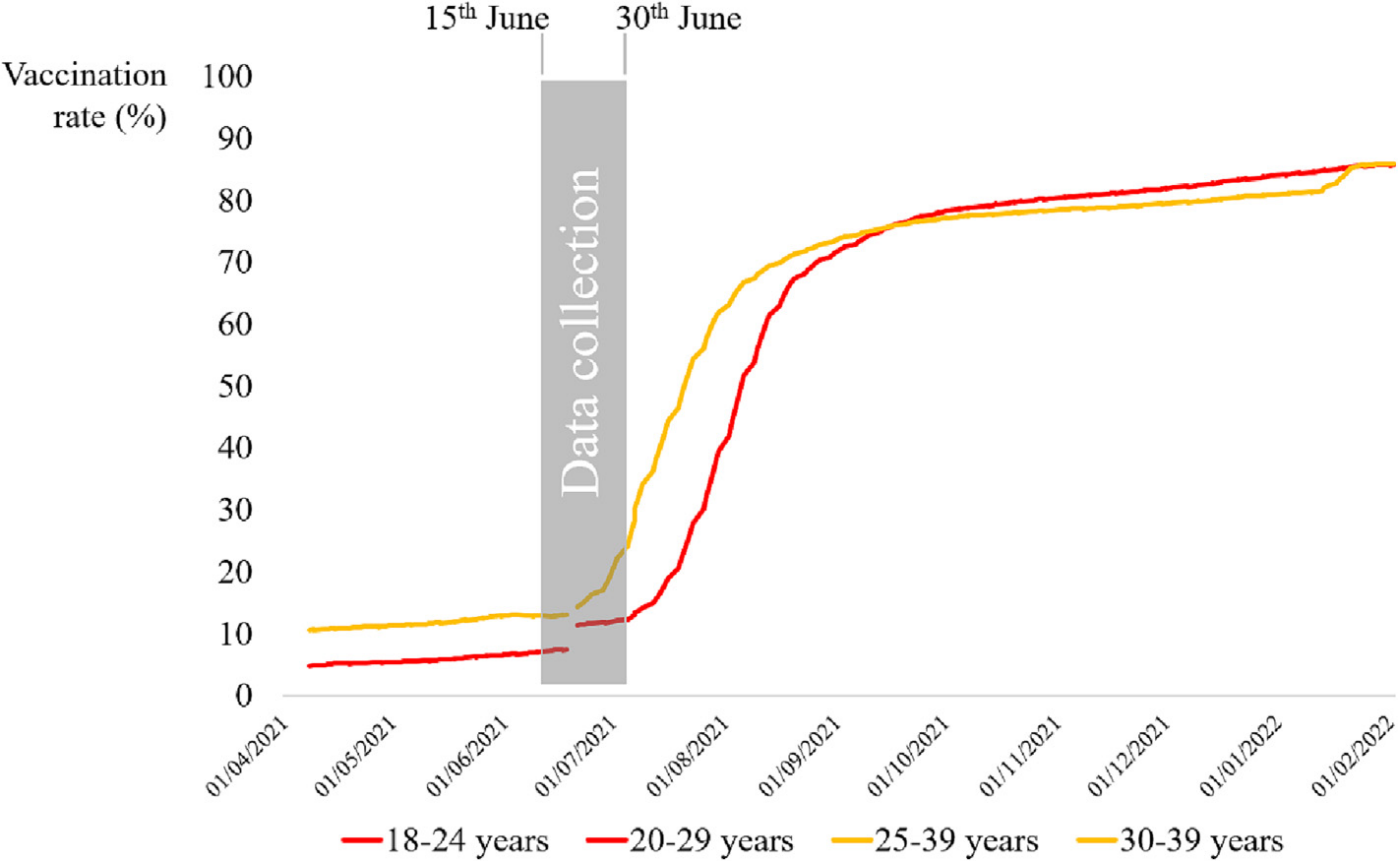
This figure shows the data collection for this study overlapped with the vaccination rate (%) of the sample of this study (first dose). The age groups are those reported by the Spanish Ministry of Health. The discontinuity seen in the graph is due to a change in the distribution criteria in age groups, which coincided with the start of general vaccination in the age range of the participants analyzed in this study. Data source: https://github.com/datadista/datasets/blob/master/COVID%2019/ccaa_vacunas_grupos_etarios_1_dosis.csv#L4659.

The study presented here is part of the PSY-COVID project. This project is an international collaborative research initiative developed during 2020 to 2022 with the purpose of generating a large database to study the psychosocial impact of the COVID-19 pandemic. A total of 180 researchers from 55 research centers in 28 countries participated, developing a standardized and cross-cultural assessment instrument for mental health, SARS-CoV-2 prevention behaviors, lifestyle, and possible predictive factors (physical, cognitive, social). Likewise, 3 waves of the study were carried out between 2020 and 2022, in which more than 92,000 people participated. This project received the approval of the *Animal and Human Experimentation Ethics Committee* of the Autonomous University of Barcelona (CEEAH-5197). The study was carried out in accordance with The Code of Ethics of the World Medical Association (Declaration of Helsinki) for experiments involving humans. This study was carried out from a cross-sectional perspective. Participants answered the PSY-COVID-2 online questionnaire anonymously from the Google Forms platform, with an approximate duration of 15 min. The design of this survey was based on the literature both on mental health and preventive behaviors. The list of variables to be measured and instruments to be included in the questionnaire was validated by a group of 30 health researchers and translated into Spanish, Catalan and English. Informed consent was included, and participation was voluntary and anonymous. This questionnaire was developed as a variant of the original PSY-COVID questionnaire with the aim of carrying out a second wave of the PSY-COVID study during 2021 and 2022. The survey was distributed through social networks (Facebook®, Instagram®, Twitter®, WhatsApp®). The form was also disseminated in 12 of the Spanish universities participating in the PSY-COVID project, including the Network of Healthy and Sustainable Universities, from June 1 to July 31, 2021, using the snowball sampling method. The dissemination campaign of the study questionnaire during the second wave was mainly addressed to young adults in Spain, coinciding with the start of the first vaccination campaign against SARS-CoV-2 for the general young adult population in this country (Fig. 1).

### 2.2 Participants

The eligibility criteria of the sample analyzed in this article were: (1) to be between 18 and 39 years old, (2) to be resident in Spain and (3) to answer the PSY-COVID-2 questionnaire between 15 and 30 June 2021. This last criterion was adopted because of the *Spanish Public Health Commission* approved on June 15, 2021, the vaccination of age groups between 12 and 39 years. Thus, the data collection period analyzed in this study is limited to the weeks of the start of the implementation of the first dose vaccination in the age range of interest. Given that an effect size equal to 1 % of the explained variance was determined according to Funder & Ozer [22] as the minimum to be detected, it was estimated that the necessary sample to extract from the database was n = 1046, for *r* = 0.1, with a type-I error of *α* = 0.05 and a type-II error of *1-β* = 0.9, relative to two-tailed Pearson bivariate correlations. Out of 2723 participants in the second wave of the PSY-COVID study, 2120 met the eligibility criteria, thus the sample size was sufficient.

### 2.3 Instruments

This study evaluated a set of 67 variables as possible predictors of intention to get vaccinated. The PSY-COVID-2 questionnaire asks participants on a) sociodemographic characteristics (age, gender, income, education), b) coronavirus impact (perceived risk, perceived vulnerability, future threats, experience of contagion; c) adaption to restrictions, d) agreement with preventive measures, e) psychological variables, f) coping strategies, g) areas of impact, and h) use of coping strategies. The psychometric properties of instruments constituted by two or more items are indicated in Table 1. The instruments of the study are detailed in the supplementary material (Table S1).

**Table 1:** Internal consistency of the instruments constituted by two items or more.

**Scale**	**Subescale**	**Cronbach's *α***	**Instrument**
Vulnerability to coronavirus		0.79	Ad-hoc
Severity		0.64	Ad-hoc
Threats		0.75	Ad-hoc
Trust	Authorities	0.82	Ad-hoc
	Experts (health staff/scientists)	0.79	
Post-traumatic growth	Post-traumatic growth	0.79	Post-traumatic Growth Inventory (PTGI-SF; Cann et al., 2010)
Depression	Depression	0.82	Patient Health Questionnaire-4 (PHQ-2; Löwe et al., 2010)
Anxiety symptoms	Anxiety	0.82	Generalized Anxiety Disorder (GAD-2; Spitzer et al., 2006)
Somatization symptoms	Somatization	0.77	Derived from systematic review. Somatization Symptoms Scale (SSQ-5; Zijlema et al., 2013)
Resilience	Recover after illness or difficulties	0.74	Connor-Davidson Resilience Scale (CD-RISC-2; Vaishnavi et al., 2007)

### 2.4 Data analysis

Statistical analysis was performed using IBM-SPSS© (Statistical Package for the Social Sciences) version 28 and was carried out on PSY-COVID-2 Dataset [24]. Descriptive statistics were extracted through measures of central tendency and dispersion (*M* and *SD*) in quantitative variables, and absolute and relative frequencies in qualitative variables. Once the scores of the different multiple-item instruments were obtained, the internal consistency was estimated.

For the purposes of some inferential statistical analysis, the original 5-points Likert scale on vaccination intention was recoded as a dichotomous variable: intention (strongly agree, agree) versus reluctance/hesitance of vaccination (neutral, disagree, strongly disagree). One-way analyses of variance were then carried out on the quantitative predictor variables for the profiling of hesitant/reluctant people in relation to their vaccination intention. With the same purpose, contingency chi-square tests were carried out in relation to dichotomous or polytomous predictor variables.

A matrix of Pearson’s product-moment bivariate correlations between the vaccination intention and its possible regressors was obtained, which allowed the identification of variables that met a double criterion: (1) a significant correlation for *α* = 0.05 and (2) a coefficient of determination (*R*^2^) greater than 1 % on the intention to vaccination. Then, a multiple regression model (stepwise method) was carried out including the variables that met these eligibility criteria as regressors of the intention to get vaccinated as the dependent variable. Finally, a discriminant analysis was carried out with the predictive variables identified in the multiple regression model to quantify their degree of sensitivity and specificity when classifying people as hesitant/reluctant about vaccination.

## 3 Results

### 3.1 Sample description

The age range of the sample (*n* = 2120) was between 18 and 39 years (*M* = 22.15; *SD* = 4.39), with a higher representation of women (*n* = 1531; 72.2 %); 97.1 % (*n* = 2058) of the participants had university studies (started or completed). Sixty percent (*n* = 1272) reported medium income, 10.8 % (*n* = 229) was health staff, 11.4 % (*n* = 242) teaching staff, and 16.3 % (*n* = 346) reported disability, mental health, or chronic illness. 11.2 % (*n* = 242) reported having passed COVID-19.

### 3.2 Profiling the vaccination hesitancy/reluctance

The comparison of means for quantitative predictors and the contingency tests for dichotomous or polytomous variables identified a total of 20 potential variables that discriminated between people with and without vaccination intention (Table 2). Belonging to health staff (*χ^2^* = 3.09; *p* =.04), perceive a future threat to own health, *F*_(1,2119)_ = 6.33; *p* =.01), tolerance of the restrictions (*F*_(1,2119)_ = 24.65; *p* <.001), having favorable attitude to mobility restrictions (*F*_(1,2119)_ = 24.65; *p* <.001), to preventive measures (*F*_(1,2119)_ = 193.67; *p* <.001) and to vaccination (*F*_(1,2119)_ = 1093.67; *p* <.001), a positive evaluation of the response of the public system facing COVID-19 crisis (*F*_(1,2119)_ = 10.19; *p* <.001), trust in authorities (*F*_(1,2119)_ = 24.65; *p* <.001) and health staff/scientists (*F*_(1,2119)_ = 271.90; p <.001), extraversion (*F*_(1,2119)_ = 6.00; *p* =.01), agreeableness (F_(1,2119)_ = 12.80; *p* <.001), time (*F*_(1,2119)_ = 18.63; *p* <.001) and quality of information about COVID-19 (*F*_(1,2119)_ = 69.10; *p* <.001), and search for social support (*F*_(1,2119)_ = 9.0.85; *p* <.001) were conditions associated with vaccination intention. Contrarily, conspiracy beliefs about SARS-CoV-2 (*F*_(1,2119)_ = 215.56; *p* <.001), availability of public resources for mental health (*F*_(1,2119)_ = 6.70; *p* =.01), good post-pandemic mood (*F*_(1,2119)_ = 3.89, *p* =.04), and coping style characterized by family support-seeking (*F*_(1,2119)_ = 10.23; *p* <.001) and substance use (*F*_(1,2119)_ = 8.58; *p* <.001) were conditions associated with hesitancy/reluctance to vaccination.

**Table 2:** Comparison between the characteristics of vaccination-intent and hesitance participants.

	**Total (*n* = 2120)**	**Hesitance (*n* = 292)**	**Intention (*n* = 1828)**	***F/χ^2^***	***p***
*Socio-demographics*					
Age: M (SD)	22.14 (4.37)	21.91 (4.43)	22.17 (4.36)	0.92^a^	0.34
Gender (female): f (%)	1523 (71.8)	209 (71.6)	1314 (71.9)	1.99^b^	0.37
Income level (medium): f (%)	1272 (60)	180 (61.6)	1092 (59.7)	0.36^b^	0.55
Education level (university): f (%)	2059 (97.1)	282 (96.6)	1777 (97.2)	0.36^b^	0.55
Health staff: f (%)	230 (10.8)	23 (7.9)	207 (11.3)	3,09^b^	**0.04**
Teaching staff: f (%)	242 (11.4)	41 (14)	201 (11)	2.31^b^	0.08
Population with disability: f (%)	20 (0.9)	5 (1.7)	15 (0.8)	2.14^b^	0.13
Population with mental health disorder: f (%)	209 (9.9)	21 (7.2)	18.8 (10.3)	2.71^b^	0.06
Population with chronic illness: f (%)	116 (5.5)	14 (4.8)	102 (5.6)	0.30^b^	0.35
Having coronavirus: f (%)	237 (11.2)	35 (12.1)	202 (11.1)	6.43^b^	0.09
A = Fisher’s *F test*; b = *Chi-square χ^2^ test*					
	**Total (*n* = 2120)**	**Hesitance (*n* = 292)**	**Intention (*n* = 1828)**	***F***	***p***

*Coronavirus impact: M (SD)*					
Perceived risk of contagion in the future (range: 0 to 4)	1.62 (0.96)	1.68 (0.96)	1.61 (0.96)	1.38	0.24
Perceived risk of contagion from others (range: 0 to 4)	1.74 (0.96)	1.84 (0.97)	1.73 (0.95)	3.64	0.06
Perceived vulnerability to coronavirus (range: 0 to 4)	1.27 (0.84)	1.28 (0.93)	1.26 (0.83)	0.15	0.70
Perceived vulnerability of others to coronavirus (range: 0 to 4)	2.03 (0.91)	2.02 (0.95)	2.04 (0.90)	0.12	0.73
Future threat: employment or studies (range: 0 to 3)	1.50 (0.97)	1.58 (0.98)	1.49 (0.96)	2.20	0.14
Future threat: income (range: 0 to 3)	1.53 (0.96)	1.57 (0.96)	1.52 (0.96)	0.46	0.50
Future threat: health (range: 0 to 3)	1.41 (0.83)	1.30 (0.82)	1.43 (0.83)	6.33	**0.01**
Future threat: personal relationships (range: 0 to 3)	1.48 (0.90)	1.49 (0.94)	1.48 (0.89)	0.02	0.88
*Adaptation to restrictions: M (SD)*					
Life changes: physical activity (range: −2 to + 2)	−0.38 (1.24)	−0.32 (1.30)	−0.39 (1.23)	0.79	0.37
Life changes: sleep habits (range: −2 to + 2)	−0.53 (1.00)	−0.53 (1.05)	−0.53 (0.99)	0.11	0.92
Life changes: diet (range: −2 to + 2)	−0.15 (0.99)	−0.13 (1.02)	−0.15 (0.98)	0.13	0.72
Life changes: income level (range: −2 to + 2)	−0.30 (0.95)	−0.36 (0.93)	−0.29 (0.95)	1.39	0.24
Life changes: work activity (range: −2 to + 2)	−0.38 (0.98)	−0.39 (0.93)	−0.38 (0.99)	0.02	0.88
Life changes: relationships with friends/family (range: −2 to + 2)	−0.22 (1.03)	−0.19 (0.99)	−0.22 (1.03)	0.31	0.58
Life changes: hobbies (range: −2 to + 2)	0.37 (1.06)	0.45 (1.04)	0.36 (1.06)	1.83	0.18
Adaptation to general changes (range: 0 to 4)	2.32 (1.00)	2.28 (0.99)	2.32 (1.00)	0.54	0.46
Time perception (range: −2 to + 2)	0.26 (1.37)	0.18 (1.39)	0.27 (1.37)	1.19	0.28
Tolerance of confinement (range: 0 to 4)	2.12 (1.25)	1.78 (1.41)	2.18 (1.22)	24.65	**< 0.001**
Leaving home during restrictions (range: 0 to 4)	2.80 (1.11)	2.85 (1.12)	2.79 (1.11)	0.61	0.43
Time spent on coronavirus information (range: 0 to 3)	0.82 (0.61)	0.61 (0.65)	0.85 (0.59)	3.11	0.08
*Agreement with preventive measures: M (SD)*					
Socioeconomics reasons virus (range: −2 to + 2)	−0.89 (1.18)	0.01 (1.26)	−1.03 (1.10)	215.56	**< 0.001**
Necessary mobility restrictions (range: −2 to + 2)	0.64 (1.12)	0.13 (1.24)	0.72 (1.07)	74.15	**< 0.001**
Necessary preventive measures (range: −2 to + 2)	1.51 (0.73)	0.98 (1.01)	1.60 (0.64)	193.67	**< 0.001**
Administration of vaccine (range: −2 to + 2)	1.62 (0.76)	0.51 (1.14)	1.80 (0.48)	1093.67	**< 0.001**
Adequate information on coronavirus (range: −2 to + 2)	−0.37 (1.09)	−0.86 (1.08)	−0.29 (1.07)	69.10	**< 0.001**
Response of the education system (range: −2 to + 2)	−0.46 (1.17)	−0.66 (1.14)	−0.43 (1.17)	10.19	**< 0.001**
Public resources for mental health (range: −2 to + 2)	−1.26 (1.11)	−1.10 (1.25)	−1.28 (1.08)	6.70	**0.01**
**Trust in government (range: 0 to 3)**	1.52 (1.34)	1.02 (1.25)	1.60 (1.33)	48.95	**< 0.001**
**Trust in health staff and scientists) (range: 0 to 3)**	4.91 (1.27)	3.84 (1.56)	5.09 (1.13)	271.90	**< 0.001**
Psychological follow-up (range: −2 to + 2)	−0.83 (1.36)	−0.92 (1.35)	−0.82 (1.36)	1.45	0.23
Change of mood post pandemic (range: −2 to + 2)	−0.73 (1.03)	−0.62 (1.10)	−0.75 (1.02)	3.89	**0.04**
*Psychological variables: M (SD)*					
Personality: extraversion (range: −2 to + 2)	0.16 (1.17)	0.00 (1.20)	0.18 (1.16)	6.00	**0.01**
Personality: conscientiousness (range: −2 to + 2)	0.47 (1.13)	0.47 (1.13)	0.47 (1.13)	0.00	0.97
Personality: agreeableness (range: −2 to + 2)	0.02 (1.19)	−0.21 (1.18)	0.06 (1.19)	12.80	**< 0.001**
Personality: neuroticism (range: −2 to + 2)	0.41 (1.13)	0.34 (1.10)	0.42 (1.13)	1.41	0.23
Personality: openness to experience (range: −2 to + 2)	0.71 (1.14)	0.65 (1.18)	0.72 (1.13)	0.89	0.34
Loneliness (range: 0 to 3)	1.20 (1.00)	1.18 (1.03)	1.21 (0.99)	0.16	0.69
Perceived competence (range: −2 to + 2)	0.91 (0.85)	0.92 (0.85)	0.91 (0.85)	0.04	0.83
Depression symptoms (PHQ-2) (range: 0 to 6)	3.03 (1.71)	3.13 (1.69)	3.01 (1.71)	1.13	0.29
Anxiety symptoms (GAD-2) (range: 0 to 6)	3.19 (1.81)	3.07 (1.80)	3.21 (1.81)	1.56	0.21
Somatization symptoms (SSQ-5) (range: 0 to 15)	4.56 (3.13)	4.57 (3.12)	4.55 (3.13)	0.01	0.92
Post-traumatic growth (PTGI) (range: 0 to 15)	6.40 (3.35)	6.51 (3.76)	6.38 (3.28)	0.37	0.54
Resilience (CD-RIS) (range: 0 to 6)	4.36 (1.08)	4.33 (1.11)	4.36 (1.80)	0.27	0.60
*Coping strategies, M (SD)*					
Hobbies (range: −2 to + 2)	−1.11 (0.79)	−1.03 (0.88)	−1.13 (0.78)	3.54	0.06
Family (range: −2 to + 2)	−0.92 (0.86)	−0.77 (0.90)	−0.95 (0.85)	10.23	**0.001**
Social activities (range: −12 to + 12)	1.95 (5.27)	1.82 (5.15)	1.97 (5.29)	0.19	0.66
Academic (range: −6 to + 6)	−3.04 (2.52)	−2.82 (2.74)	−3.07 (2.48)	2.11	0.15
Work impact (range: −2 to + 2)	−0.39 (0.85)	−0.44 (0.88)	−0.38 (0.84)	1.47	0.22
Hospitalization (range: −2 to + 2)	−0.43 (0.84)	−0.40 (0.84)	−0.43 (0.84)	0.42	0.51
Health habits (range: −6 to + 6)	−0.66 (2.33)	−0.59 (2.34)	−0.67 (2.33)	0.30	0.58
Information about coronavirus (range: −2 to + 2)	−0.05 (0.89)	−0.27 (0.87)	−0.02 (0.89)	18.63	**< 0.001**
Technological activities (range: −2 to + 2)	0.36 (0.96)	0.35 (0.99)	0.36 (0.96)	0.03	0.96
Use of social media (range: −2 to + 2)	0.12 (1.12)	0.04 (1.10)	0.13 (1.12)	1.54	0.21
Meet with friends (range: −2 to + 2)	0.29 (1.44)	0.17 (1.43)	0.31 (1.44)	2,01	0.16
*Use of coping strategies, M (DE)*					
Focus on coping with adversity (range: 0 to 3)	1.38 (0.80)	1.38 (0.79)	1.38 (0.81)	0.00	0.98
Substance use (range: 0 to 3)	0.43 (0.76)	0.55 (0.89)	0.41 (0.74)	8.58	**< 0.01**
Express feeling bad (range: 0 to 3)	1.55 (0.87)	1.50 (0.94)	1.56 (0.86)	1.28	0.26
Seek emotional support (range: 0 to 3)	1.58 (0.91)	1.43 (0.95)	1.61 (0.89)	9.85	**< 0.01**
Denial (range: 0 to 3)	0.33 (0.65)	0.37 (0.72)	0.32 (0.63)	1.16	0.28
Joke about circumstances (range: 0 to 3)	1.66 (1.02)	1.62 (1.00)	1.67 (1.02)	0.63	0.43
Seek help from God (range: 0 to 3)	0.21 (0.61)	0.27 (0.68)	0.21 (0.59)	2.49	0.11

### 3.3 Predictive modeling of vaccination intention

In order to perform the linear multiple regression modeling of the vaccination intention, a Pearson’s product-moment correlation matrix was carried out (see Supplemental Table S2). A total of 35 variables met criterion 1 (a significant correlation for *α* = 0.05), and a total of 8 variables additionally met criterion 2 (coefficient of determination *R*^2^ greater than 1 % adopted according to Funder & Ozer statement about the smaller effect (*r* = 0.10) that has potential to be consequential [22]). These variables ordered by effect size were attitude to vaccination (*r* = 0.62; *p* <.001), trust in health staff/scientists (*r* =. 37; *p* <.001), conspiracy beliefs about SARS-CoV-2 (*r* = 0.34; *p* <.001), attitude towards non-pharmacological preventive measures (*r* = 0.31; *p* <.001), time spent to get informed about COVID-19 (*r* = 0.20; *p* <.001), attitude to mobility restrictions (*r* = 0.20; *p* <.001), trust in authorities (*r* = 0.15; *p* <.001) and adaptation to mobility restrictions (*r* = 0.12; *p* <.001). Then, the multiple linear regression model was performed with the stepwise procedure (Table 3), in which the vaccination intention was introduced as a dependent variable and the eight variables that met the two eligibility criteria indicated above were included as regressors. The results indicated that a model with 4 regressors showed a multiple correlation of *r* = 0.65 and thus a predictive capacity (*R*^2^_adjusted_) of 41 % (*F*_(1,2111)_ = 500.95; *p* <.001): Attitude to vaccination, trust in health staff/scientists and time of information about COVID-19 showed positive *β* scores, but conspiracy beliefs about SARS-CoV-2 showed a negative *β* score.

**Table 3:** Multiple linear regression for the intention to get vaccinated.

	***β***	***t***	**Change in adjusted *R^2^***	**Adjusted** ***R^2^***	***F***
**Intention to vaccinate**				0.41	365.35***
Attitude to vaccine	0.52	27.11***	0.37		
Trust in health staff and scientists	0.13	6.83***	0.02		
Conspiracy beliefs about SARS-CoV-2	-0.11	−6.26***	0.01		
Time spent in coronavirus information	0.03	1.98*	0.01		

* p <.05; ** p <.01; ***p <.001.

### 3.4 Discriminant analysis of vaccination intention

Once the set of variables with predictive capacity of the vaccination intention was identified, a discriminant analysis was performed. To this end, a dichotomous classification derived from the vaccination intention scale was adopted, which involves the distinction between people who reject or doubt their intention to get vaccinated (13.9 %) from those with a vaccination intention (86.1 %). A robust discriminant function was obtained as a result (intention centroid: 0.31; non-intention centroid: −1.89; Wilk's *λ* = 0.63, *r*_canonic_ = 0.64; *p* <.001), which allowed the correct classification of 86 % of cases (sensitivity = 89 %; specificity = 70 % for the intention to get vaccinated against the SARS-CoV-2 virus) based on three predictors: conspiracy beliefs about SARS-CoV-2, attitude on population vaccination and time spent to be informed about COVID-19.

## 4 Discussion

The purpose of this study was to identify the main predictors of vaccination intention in a population with high hesitation/reluctance in a country with a high vaccination rate. Firstly, it was found that 86 % of the people in this study (Spanish young adults) expressed intention to get vaccinated against SARS-CoV-2. It is known that Spain is one of the countries in Europe with the highest acceptance of vaccination [25], probably because it was one of the countries most affected by this virus in the first wave in early 2020, with more than 100,000 deaths and 11.5 million of cases confirmed so far. At a time when it seems to be necessary to reach at least 90 % of vaccinated to achieve group immunity [6] and considering that there is a high degree of the population that for various reasons (age, chronic diseases, etc.) cannot be vaccinated, it is necessary to ensure the maximization of the intention to get vaccinated by the rest of the population [[4], [26]].

Secondly, the results identify a pool of heterogeneous conditions from different domains related to the intention of getting vaccinated. Belonging to health staff, perceiving a threat to own health, high tolerance of confinement, favorable attitude to mobility restrictions, to preventive measures and to the vaccine, trust in the authorities and in health staff/scientists, confidence in the public system, personality (extraversion and agreeableness), time and quality of information about COVID-19 and seeking emotional support were conditions associated with the intention to get vaccinated. In contrast, conspiracy beliefs about SARS-CoV-2, availability of public resources for mental health, good post-pandemic mood, and coping style characterized by seeking family support and substance use were conditions associated with hesitancy/reluctance to vaccination.

Within the broad pool of variables analyzed, the model that best predicts intention to get vaccinated includes four variables accounting for 41 % of the interindividual variability: attitude toward pharmacological measures for SARS-CoV-2, trust in experts (scientists and health professionals), conspiracy beliefs about SARS-CoV-2 and time expend information. Moreover, the discriminant analysis showed that by asking just three questions, it is possible to identify, with an accuracy of 86 %, young adults who are reluctant or hesitant about vaccination: 1) “Is it necessary to administer the vaccine to the population?” (Attitude towards pharmacological measures against COVID-19); 2) “What degree of trust have scientists and health professionals deserved during the coronavirus crisis?” (Trust in health staff/scientists) and 3) “do you think SARS-CoV-2 is a virus created for socioeconomic purposes”? (Conspiracy beliefs about SARS-CoV-2).

Our findings are in line with previous literature that has highlighted that to believe that the vaccine is needed in the population to eradicate the virus strongly predicts the intention to get vaccinated. Various studies have shown that the attitude to population vaccination to face COVID-19 disease is closely linked to the personal intention to get vaccinated [[27], [28]]. Lugo-González [29] found that positive beliefs and attitudes regarding vaccination are one of the most relevant psychosocial factors in the process of immunizing the population against SARS-CoV-2.

Like our study, lack of trust in authorities in general, and in science in particular, has been found to be a major predictor of various conspiracy theories [[30], [31]] mediating the negative effect of conspiracy theories on the level of adherence to health indications [[32], [33]]. Cavojova [34] found that preventing the spread of conflicting scientific knowledge facilitates greater understanding and belief in science, directly influencing people's intention to get vaccinated. The diversity of scientific discourses, showing different points of view on the same object of study, affects the level of trust they inspire in the population [34]. This phenomenon responds to the usual functioning of science, which does not necessarily follow linear patterns [[35], [36]]. However, the level of uncertainty and the need for immediate responses from both the health system and citizens, has meant that all eyes are on their discourse [37]. Since the outbreak of SARS-CoV-2, confidence in health institutions and in the opinions of health experts has been weakening [[38], [39]], resulting in lower adherence to essential health recommendations and contributing to the transmission of the virus [[38], [40]].

In a context of great uncertainty and little precedent, the need to cling to as much information as possible is understood. However, the lack of validated information and the dissemination of false news are two of the main reasons for the growing doubt about vaccination [[41], [42]]. While fake news has always existed, its spread in the digital age is growing exponentially and making it difficult to control [43]. Dubè [44] argues that false news is one of the main factors that explain the low level of immunity. Within the dissemination of fake news, the effect of the dissemination of conspiracy theories has had a clear negative effect on the intention to get vaccinated [[45], [46]]. This effect had also been previously observed with respect to non-pharmacological prevention measures against SARS-CoV-2.

As a synthesis, it is worthy to be highlighted that these main predictive factors of reluctance or hesitation about vaccination (vaccine attitude, trust on key actors, conspiracy beliefs, and information on COVID-19) are (1) of a social nature and (2) modifiable through the adoption of effective communication strategies, related to trust in key groups, attitude towards vaccination, information of quality about the pandemic and prevention measures (pharmacological and non-pharmacological) and the control of fake news. Therefore, they must be at the core of public health policies in future pandemics.

Not all the results obtained in this study are in line with the findings of the previous literature. Surprisingly and contrary to our findings, most studies report that women show a lower intention of vaccination against SARS-CoV-2 [[47], [48]], contrary to what has been observed in terms of non-pharmacological measures to prevent the virus [[49], [50]]. It has been argued that this may be due to a gender gap with respect to lower risk-taking by women, as well as being more proactive about preventative behaviors compared to men [[51], [52]]. This, together with the lack of evidence in our study (contrary to that indicated by other previous studies) regarding the relationship between various sociodemographic factors and vaccination intention, may be related to the particularities of the study population, which is discussed in greater detail later.

### 4.1 Strengths and limitations

The main strength of this study is that its results allow an explorative analysis of a wider variety of variables associated with reluctance to vaccination (compared to studies that focus on a narrow spectrum of probable predictive variables) which leads to a comprehensive model. The diversity of the pool of variables collected, which includes affective, cognitive, contextual and sociodemographic factors, has made it possible to understand the heterogeneity of the processes behind the decision to vaccinate or not against SARS-CoV-2.

Another important strength of this study is that the date of dissemination of the questionnaire coincided exactly with the time of access to the vaccine for the age range of 18 to 39 years, which was the population of interest for this study because it was the most resistant to vaccination.

Among the limitations we highlight the level of homogeneity of the sample. The sample included in the study was highly specific and showed relevant biases (97 % had a university degree in progress or completed, and 72 % were female) that must be considered with regard to the generalizability of the findings of this study. Regarding the gender bias of the representativeness of the sample, this limitation would have been a major problem if the gender variable had appeared as a variable with great explanatory power for the intention to vaccinate. In any case, this may explain why no significant correlations were found between sociodemographic variables and vaccination intention, contrary to findings in previous literature. Concretely, it has been observed that having a high level of education is a strong predictor of the intention to get vaccinated [[15], [53]], associated with greater access and processing of vaccination information received regarding the vaccine and its effects.

However, a bias in the sample of this study (predominance of people with a university level with access to online media) endows an important learned lesson: even in a population characterized by a high level of education and access to information, which presupposes access to quality information and capacity for critical analysis, the conspiracy ideas derived from fake news and the lack of information can have a robust influence on the intention to get vaccinated.

## 5 Conclusions

We conclude that this cross-sectional research makes it possible to identify, within a wide and heterogeneous range of variables evaluated, the factors that best predict the intention of vaccination among young adults in Spain in the beginning of their vaccination campaign: Attitude to vaccination, trust in health staff/scientists, time of information and conspiracy beliefs about SARS-CoV-2. Also, this study has made it possible to delineate in detail the profile of people with high doubts or rejection of vaccination.

## Funding

This study has been funded by the Agency for Management of University Research Grants (AGAUR; 2020PANDE00025) of the Government of Catalonia, the Institute of Health Carlos III (ISCIII; ICI20/00080) and has been co-financed with European Union ERDF funds. JPS-M has a PFIS predoctoral contract from the ISCIII (FI20/00034). AF-S acknowledges the funding from the Serra Húnter program (UAB-LE-8015).

The sources of funding are public agencies responsible for the management of funds allocated through government budgets to competitive calls and does not participate in the design of research.

## Data Availability Statement

The data supporting the findings of this study are available on Figshare: https://figshare.com/articles/dataset/PSY-COVID-2_raw/20045783.

## Statement of the Institutional Review Committee

This study was approved by the Ethics Committee for Animal and Human Experimentation of the Autonomous University of Barcelona (CEEAH-5197), following the guidelines of the Declaration of Helsinki.

## CRediT authorship contribution statement

**Corel Mateo-Canedo:** Conceptualization, Validation, Investigation, Resources, Data curation, Writing – original draft, Writing – review & editing, Visualization. **Juan Pablo Sanabria Mazo:** Conceptualization, Validation, Writing – original draft, Writing – review & editing. **Laura Comendador:** Conceptualization, Validation, Investigation, Resources, Data curation, Writing – original draft, Writing – review & editing. **Juan Sebastián Rojas:** Validation, Investigation, Resources, Data curation, Writing – review & editing. **Meritxell Carmona:** Validation, Investigation, Resources, Data curation, Writing – review & editing. **Neus Crespo Puig:** Validation, Investigation, Resources, Writing – review & editing. **Fiorella Anyosa:** Validation, Investigation, Resources, Writing – review & editing. **Clara Selva:** Validation, Investigation, Resources, Writing – review & editing. **Albert Feliu-Soler:** Validation, Investigation, Resources. **Narcís Cardoner:** Validation, Investigation, Resources, Writing – review & editing. **Juan Deus:** Validation, Investigation, Resources, Writing – review & editing. **Juan V. Luciano:** Validation, Resources, Funding acquisition. **Jorge Luis Méndez-Ülrich:** Validation, Investigation, Resources, Writing – review & editing. **Antoni Sanz:** Conceptualization, Methodology, Validation, Formal analysis, Investigation, Resources, Data curation, Writing – original draft, Writing – review & editing, Visualization, Supervision, Project administration, Funding acquisition.

## Declaration of Competing Interest

The authors declare that they have no known competing financial interests or personal relationships that could have appeared to influence the work reported in this paper.
