# Supplementary material for: Predictive factors of hesitancy to vaccination against SARS-CoV-2 virus in young adults in Spain: Results from the PSY-COVID study
Source: Vaccine X. 2023 Apr 18;14:100301. doi: 10.1016/j.jvacx.2023.100301 (PMC10112940; doi:10.1016/j.jvacx.2023.100301)
Supplement: Supplementary data 1 [file mmc1.pdf]

[Supplemental material]

Table S1. List of items (see instruments description below)

Variable (role)	*Scale*	*Statement of section*	*Statement of question*	*Item*	*Labels*	*Origin of the scale*
Behavioral changes	Ordinal 5 bipolar	The following questions are related to the period in which restricted mobility measures have been adopted to control the spread of the coronavirus in the place where you have been living	Indicate to what extent have changed the following aspects of your life:	Physical activity	Much worse, Worse, The same, A little better, Much better	Ad-hoc
				Sleep Habits		
				Diet		
				Income level		
				Work activity		
				Relationships with your partner or your family		
				Relationships with your neighbors		
				Relationships with friends and people you know		
				Hobbies (reading, video games, music, cooking, etc.)		
Post-traumatic growth: relating to others	Ordinal 4		Please indicate to what extent you have experienced the following changes during the pandemic:	I have felt closer to others	Not at all, A little, Quite a lot, A lot	Post-traumatic Growth Inventory (PTGI-SF; Cann et al., 2010)
Post-traumatic growth: new possibilities				I have felt more capable of improving my life		
Post-traumatic growth: personal strength				I have discovered that I am stronger than I thought		
Post-traumatic growth: spiritual change				I have gained a deeper understanding of spiritual matters		
Post-traumatic growth: appreciation of life				I now appreciate the value of my own life much more		
Adaptation to mobility restrictions	Ordinal 5 bipolar		How have you adapted to the changes in your life?		Very badly, Badly, Neither well nor badly, Well, Very well	Ad-hoc
			How have people around you adapted to the changes in their lives?			
			Would you say that time has passed slowly or quickly?		Very slowly, Fairly slowly, Fairly quickly, Very quickly	
Tolerance of confinement	Ordinal 5		How much longer do you think you could deal with the restrictions?		No longer, A few more days, A few more weeks, A few more months, Indefinitely	
Restriction level	Ordinal 5		How often, approximately, have you left home?		Never, Once a month, Every 15 days, Once a	Ad-hoc

					week, Once a day	
	Ordinal 5 bipolar		How much have you been out of the house in relation to what was usual for you?		Much less, Somewhat less, The same, More, Much more	
Mood prior to the pandemic	Ordinal 5 bipolar		How do you think is your mood in relation to what it was before the pandemic?		Much worse, Worse, The same, A little Better, Much better	Ad-hoc
Coping strategies: active coping and planning	Ordinal 5		Indicate how often you have been using the following strategies to cope with adversity along to the pandemic:	I focus on dealing with this problem, and if necessary, let other things slide a little	Not at all, A Little, Quite a lot, A lot	Ad-hoc
Coping strategies: use of alcohol or drugs				I drink alcohol or take drugs, in order to think about it less		
Coping strategies: emotional ventilation				I feel a lot of emotional distress and I find myself expressing those feelings a lot		
Coping strategies: social support				I try to get emotional support from friends or relatives		
Coping strategies: denial				I refuse to believe that it has happened		
Coping strategies: humor				I kid around about it		
Coping strategies: religion				I seek God’s help		
Psychological follow-up	Dichotomous		Are you currently receiving psychological/psychiatric follow-up?		No, not me I raised, No, but I'm thinking, Yes, before the pandemic, Yes, since the pandemic began	Ad-hoc
Trust	Ordinal 5	The following questions refer to the present:	Indicate the degree of trust that the following have earned during the coronavirus crisis:	The ruling bodies of your country	None, A little, Quite a lot, A lot	Ad-hoc
				The ruling bodies of your region		
				People in general		
				Health staff		
				Scientists		
Information level	Ordinal 4		Indicate the amount of time that you spend focusing on information related to the coronavirus during the day:		Zero, Less than 1h, 1h-2h, 3h or more	Ad-hoc
Attitudes	Likert 5		Please indicate your agreement or disagreement with the following statements:	SARS-CoV-2 is a virus created for socio-economic purposes	Strongly disagree, Disagree, Neutral, Agree, Strongly agree	Ad-hoc
				Mobility restrictions imposed are necessary		
				Prevention measures (distance, handwashing, mask ventilation) are required		
				It is necessary to administer the vaccine to the population		

				It is well-informed people about the SARS-CoV-2 virus		
				The public system (health, education) has adapted to changes		
Opinion	Likert 5			Public resources are sufficient to promote good mental health	Strongly disagree, Disagree, Neutral, Agree, Strongly agree	Ad-hoc
Vulnerability to coronavirus	Ratio 0-100	The following questions are about the next weeks	In your view, what is:	The likelihood that you will become infected with coronavirus in the future?	Highly unlikely, Unlikely, Moderate, Likely, Highly likely	Ad-hoc
				The likelihood of people around you becoming infected with coronavirus?		
Severity	Ordinal 5		In your view, what would be:	The severity of the symptoms you would experience if you became ill in the future?	Very mild, Mild, Moderate, Severe, Very severe	Ad-hoc
				The severity of the symptoms that people around you would experience if they became ill in the future?		
Future threat	Ordinal 4		To what extent do you feel that the following aspects of your life are under threat in the immediate future:	Your employment or studies	Not at all, A little, Fairly, Very much	Ad-hoc
				Your income		
				Your health		
				Your personal relationships		
Intention to vaccinate	Likert 5		Do you agree to be vaccinated against SARS-CoV-2?		Strongly disagree, Disagree, Neutral, Agree, Strongly agree	Ad-hoc
Depression symptoms	Ordinal 4	In the last two weeks, indicate how often ...	You have experienced:	Little interest or pleasure in doing things	Never, Sometimes, More than half of the time, Almost every day	Patient Health Questionnaire-4 (PHQ-2; Löwe et al., 2010)
				Weariness, depression, or hopelessness		
Anxiety symptoms	Ordinal 4			Nervousness or tension		Generalized Anxiety Disorder (GAD-2; Spitzer et al., 2006)
				Inability to stop or control worrying thoughts		
Loneliness	Ordinal 4			Loneliness		UCLA Loneliness Scale (UCLS; Russell et al., 1978)
Somatization symptoms	Ordinal 4			Headache		Derived from systematic review. Somatization Symptoms Scale (SSQ-5;
				Nausea or stomach pain		
				Sensation of breathlessness		
				Dizziness		
				Back pain		

						Zijlema et al., 2013)
Resilience	Ordinal 4	The following questions do not refer to a specific time, but how you feel in general:	To what extent do you feel able to:	Recover after illness or difficulties	Unable, Slightly able, Quite able, Very able	Connor-Davidson Resilience Scale (CD-RISC-2; Vaishnavi et al., 2007)
				Adapt to changes		
Personality: extraversion	Likert 5		Indicate to what extent the following statements apply to you:	I like having lots of people around	Strongly disagree, Disagree, Neutral, Agree, Totally agree	NEO Five-Factor Inventory (NEO-FFI; Manga et al., 2004)
Personality: conscientiousness				I have a lot of self-discipline		
Personality: agreeableness				My first reaction is to trust people		
Personality: neuroticism				I often feel tense or anxious		
Personality: openness to experience				I'm curious about the forms I find in art and nature		
Perceived competence				Overall, I am able to achieve the things I want		Perceived Competence Scale (PCS; Fernnández-Castro et al., 1998).
Population cluster	Nominal	Socio Demographic questions	Indicate if you belong to any of these groups (you can check more than one)	Health staff		Ad-hoc
				Teaching staff		
				Person with disability		
				Person with mental health disorder		
				Person with chronic illness		
Country	Nominal			Country of residence during the period of restrictions	According to language	Ad-hoc
Region	Nominal			Region of residence during the period of restrictions	According to country	Ad-hoc
Education	Ordinal 4			Level of education	Without studies, Primary / basics, Secondary, University	Ad-hoc
Socio-economic status	Ordinal 3			Income level	Low, Medium, High	Ad-hoc
Gender	Nominal			Gender	Female, Male, Other	Ad-hoc
Age (predictive)	Ratio			Age	16-99 or older	Ad-hoc
Coronavirus infection	Ordinal 4		(OPTIONAL) Have you caught coronavirus?		Definitely, Probably, Probably not, Definitely not	Ad-hoc
			(OPTIONAL) Has someone close to you died or suffered from a		Yes, No	

			severe form of the disease?			
University community	Nominal		(OPTIONAL) Do you belong to a university community?	List of universities according to country		Ad-hoc
			If you belong to a university community, indicate to which specific group:		I do not belong to a university community, Student, Teaching and Research Staff, Administration and Services Staff	Ad-hoc
Level of study (predictive)	Ordinal	Student	(If student) Indicate the studies currently enrolled		Degree, Master/postgraduate, Doctorate	Ad-hoc
Impact factors (mental health)	Ordinal		From the following activities or situations, please indicate to what extent you have been benefited or harmed to cope to adverse situations:	Unable to perform common hobbies<br>Not being able to relate to my family<br>Not being able to relate to my friends<br>Not having a college classroom experience<br>Take online classes<br>Do academic homework<br>Unemployment or modification of labor contract<br>Loss or hospitalization of loved ones<br>Sleeping habits<br>Healthy eating habits<br>Basic self-care (showering, dressing in clean clothes, etc.)<br>Informing yourself about the COVID-19 pandemic<br>Practice creative activities such as writing, painting, music, cooking, etc.<br>Technological practice activities such as surfing the Internet<br>Sport the outdoors including hiking, running, mountain<br>Activities such as walking, dog-walking, or shopping<br>Practice indoor activities like dancing, team sports<br>Using social networks like Instagram, or Twitter Tik-Tok<br>Meet friends to go drinking, go to dinner, etc.<br>Go to cultural events like movies, concerts, theater	Affected me a lot, Affected me something, Neutral, Benefited me something, Benefited me a lot	Ad-hoc

Emotional support demand (mental health)	Dichotomous	Do you think It would be useful to have a platform where you could manage emotions, as well as spaces for interaction with professionals in mental health?	Yes, No	Ad-hoc

Instruments' details

Socio-demographic variables
Age, gender, income, and education level were measured with an *ad-hoc* inventory.

Coronavirus impact
Several *ad-hoc* inventories were developed to assess the impact of the COVID-19 pandemic, such as de perceived risk and vulnerability during the period of restrictions, the perceived future threat in social, academic, economic and health areas and the experience of contagion in oneself or close people. The answer to the items was through ordinal scales of 5 or 4 labels.

Adaption to restrictions
Through several ordinal scales of 5 or 4 labels it was evaluated the changes in different life aspects like physical activity, sleep habits and diet, the adaptation to general changes during the pandemic restrictions, the time perception, the tolerance of confinement, the frequency of leaving home and the time spent on coronavirus information.

Agreement with preventive measures
A five items *ad-hoc* inventory was used to measure the level of trust in social agents critical in the management of the pandemic. According to the consensus reached by the panel of experts who developed the PSY-COVID study, these agents were the country's authorities, regional authorities, health staff, scientists and the general population. The answer to this inventory was a 4-point Likert-type scale, in which 0 corresponds to "nothing" and 3 to "much". Higher values indicate greater trust. An exploratory factorial analysis revealed that the five items of this instrument were grouped in three subscales: Authorities (country and regional), experts (health staff and scientists) and general population. The two-item subscales of this inventory had adequate internal consistency in this sample: trust in authorities (Cronbach's $\alpha = .82$) and trust in health staff/scientists (Cronbach's $\alpha = .79$).
An *ad-hoc* inventory of 6 items was developed to assess attitudes in relation to causes and effects of the pandemic: conspiracy belief about the origin of the virus, adequacy of the mobility restrictions, pharmacological (vaccine) and non-pharmacological measures against SARS-CoV-2, information to the population, and response of public services (health, education) facing to the pandemic. The answer to this item was a 5-point Likert type, in which -2 corresponds to "strongly disagree" and +2 to "strongly agree". As expected, an exploratory factorial analysis revealed the lack of a factor structure, thus each item was analyzed separately.

Psychological variables
NEO Five-Factor Inventory (NEO-FFI; Manga et al., 2004) was used to measure the five dimensions of personality (neuroticism, extraversion, agreeableness, sensation seeking, and openness to experience). The highest factor saturation item for each dimension was used in this study. The answer to this item was a

5-point Likert type, in which -2 corresponds to "strongly disagree" and +2 to "strongly agree". Higher values indicate greater traits in each dimension of the personality.

UCLA Loneliness Scale (UCLS; Russell et al., 1978) was used to measure the feeling of loneliness-trait. The highest factor saturation item of the instrument was used in this study. The answer to this item was 4-point Likert type, with 0 corresponding to "no day" and 3 to "almost every day". Higher values indicate a greater sense of loneliness.

Perceived Competence Scale (PCS; Fernández-Castro et al., 1998) was used to measure perceived competence. The highest factor saturation item of the instrument was used in this study. The answer to this item was a 5-point Likert type, in which -2 corresponds to "strongly disagree" and +2 to "strongly agree". Higher values indicate more perceived competence.

Patient Health Questionnaire (PHQ-2; Löwe et al., 2010) was used to measure symptoms of depression. This abbreviated version contains 2 items with a 4-point Likert-type response format, in which 0 corresponds to "no day" and 3 to "almost every day". The total score ranges from 0 to 6, with the cut-off point for detecting clinically relevant symptoms of depression equal to or greater than 3. PHQ-2 reported adequate internal consistency (Cronbach's α = .82) in this sample.

Generalized Anxiety Disorder (GAD-2; Spitzer et al., 2006) was used to measure anxiety symptoms. This abbreviated version contains 2 items with a 4-point Likert-type response format, in which 0 corresponds to "no day" and 3 to "almost every day". The total GAD-2 score ranged from 0 to 6, with the cut-off point for detecting clinically relevant anxiety symptoms equal to or greater than 3. GAD-2 reported adequate internal consistency (Cronbach's α = .83) in this sample.

Somatization Symptoms Scale (SSQ-5; Zijlema et al., 2013) was used to measure somatization symptoms. It consists of the most frequent five items identified in the systematic review of Zijlema et al. (2013) about somatic symptoms assessment tools. This instrument is evaluated in a 4-point Likert-type response format, in which 0 corresponds to “no day” and 3 to “almost every day”. The total SSQ-5 score ranged from 0 to 15. This instrument reported adequate internal consistency (Cronbach's α = .77) in this sample.

Posttraumatic Growth Inventory-Short Form (PTGI-SF; Cann et al., 2010) was used to measure posttraumatic growth. The version of this instrument adapted for the PSY-COVID questionnaire contains 5 items. For this abbreviated version, the structure of 5 second-order factors of the general scale was considered (relating to others, new possibilities, spiritual change, appreciation of life) and the item that showed the greatest factorial saturation with each of them was chosen. This construct was evaluated with a 4-point Likert-type response format, in which 0 corresponds to “nothing” and 3 to “a lot”. The total score ranges from 0 to 15, indicating the highest values increased post-traumatic growth. This brief version of PTGI-SF reported adequate internal consistency (α = .79) in this sample.

The Connor-Davidson Resilience Scale (CD-RISC-2; Vaishnavi et al., 2007) was used to measure resilience. This instrument contains 2 items with a 4-point Likert-type response format, in which 0 corresponds to “nothing” and 3 to “a lot”. The total score ranges from 0 to 6, indicating the highest values greater resilience. CD-RISC-2 showed a limited internal consistency (Cronbach's α = .65) in this sample.

Use of coping strategies

Brief-COPE (Carver, 1997) was the basis for the development of an adapted version of the PSY-COVID questionnaire for the evaluation of coping strategies. In accordance with the panel of experts that developed the second wave version of the questionnaire, the items with the highest factorial saturation were selected from the subscales related to the seven coping strategies that could be most relevant in relation to the management of the pandemic: active coping, alcohol or drug use, emotional ventilation, social support, denial, humor and religion. Responses were given on a Likert-type scale from 0 "Not at all" to 3 "A lot".

Attitude towards vaccination

Intention to get vaccinated, the main outcome of this study, was evaluated with the single item “Do you agree to get vaccinated against SARS-CoV-2?” in a 5-point Likert scale ranging from “strongly disagree” to “strongly agree”.

Table S2

Pearson's product-moment correlations between the intention to vaccinate and the predictor variables

	**Intention to vaccinate**
**Socio-demographics**	
Gender	.00
Age	.01
Income level	.02
Education level	.03
**Coronavirus impact**	
Having coronavirus	-.06**
Near death or suffering from coronaviruses	-.01
Perceived risk of contagion in the future	-.06**
Perceived risk of contagion from others	-.06**
Perceived vulnerability to coronavirus	-.05*
Perceived vulnerability of others to coronavirus	-.01
Future threat: employment or studies	-.04*
Future threat: personal economy	-.04*
Future threat: health	.04*
Future threat: personal relationship	-.01
**Adaptation to restrictions**	
Life changes: physical activity	-.02
Life changes: sleep habits	-.01
Life changes: diet	-.02
Life changes: income level	.04
Life changes: work activity	.02
Life changes: relationships with friends/family	-.01
Life changes: hobbies	-.04
Adaptation to general changes	.02
Time perception	.03
Tolerance of confinement	**.12*** **
Leaving home during restrictions	-.04*
Time spent on coronavirus information	**.13*** **
**Agreement with preventive measures**	

Socioeconomics reasons virus	**-.34*** **
Necessary mobility restrictions	**.19*** **
Necessary preventive measures	**.30*** **
Administration of vaccine	**.61*** **
Adequate information on coronavirus	**.20*** **
Response of the education system	.05*
Public resources for mental health	-.06*
**Trust in government**	**.14*** **
**Trust in experts (health staff and scientists)**	**.37*** **
Psychological support	.02
Platform utility to manage emotions	.07**
Post-pandemic change of mood	-.05*
**Psychological variables**	
Personality: extraversion	.04
Personality: conscientiousness	-.01
Personality: amability	.07**
Personality: neuroticism	.02
Personality: openness to experiences	.00
Loneliness	.02
Perceived competence	-.01
Depression symptoms (PHQ-2)	-.02
Anxiety symptoms (GAD-2)	.03
Somatization symptoms (SSQ-5)	-.01
Post-traumatic growth (PTGI)	-.05*
Resilience (CD-RISC)	.01
**Coping strategies**	
Hobbies	-.05*
Family	-.05*
Social activities	-.01
Academic	-.04
Work impact	.03
Hospitalization	-.02
Health habits	-.02
Information about coronavirus	.09***
Technological activities	.03

Use of social media	.05*
Meet with friends	.03
**Use of coping strategies**	
Focus on coping with adversity	-.01
Substance use	-.06**
Express feeling bad	.02
Seek emotional support	.06**
Denial	-.05*
Joke about circumstances	-.03
Seek help from God	-.05*

Note: Values are Pearson's correlations. * $p < .050$; ** $p < .01$; ***$p < .001$. Variables included in the linear multiple regression model are indicated in bold.
